# Co‐Designing a Digital Stroke Prevention Platform: Leveraging Lived Experience and Expert Advice

**DOI:** 10.1111/hex.70293

**Published:** 2025-05-22

**Authors:** Tara Purvis, Catherine Burns, Seamus Barker, Monique F. Kilkenny, Seana L. Gall, Christine Farmer, Vaishnavi Sudhakar, Dominique A. Cadilhac, Brenda Booth, Janet E. Bray, Jan Cameron, Lachlan L. Dalli, Stephanie Ho, Eleanor Horton, Timothy Kleinig, Lisa Murphy, Mark R. Nelson, Muideen T. Olaiya, Amanda G. Thrift, Rosanne Freak‐Poli

**Affiliations:** ^1^ Stroke and Ageing Research, Department of Medicine, School of Clinical Sciences at Monash Health Monash University Clayton Melbourne Australia; ^2^ Menzies Institute for Medical Research University of Tasmania Hobart Australia; ^3^ Stroke Theme, Florey Institute of Neuroscience and Mental Health University of Melbourne Melbourne Australia; ^4^ Stroke Foundation Melbourne Australia; ^5^ School of Public Health and Preventive Medicine Monash University Melbourne Australia; ^6^ Department of Neurology Royal Adelaide Hospital Adelaide Australia; ^7^ Department of Medicine University of Adelaide Adelaide Australia

**Keywords:** co‐design, consumer engagement, digital health, primary prevention, stroke, technology

## Abstract

**Background:**

The majority of strokes are preventable through effective risk factor management. Existing primary prevention strategies have insufficient reach and effectiveness. Digital health technologies offer the potential to overcome some of these barriers. The aim of this study was to co‐design the ‘Love Your Brain’ digital platform, including an online education program (Massive Open Online Course, MOOC) and text messaging system, for community stroke prevention education and management.

**Methods:**

Using snowballing methods, expressions of interest were sought from community members and health knowledge experts (e.g., health professionals and researchers) from across Australia. Participants were purposively selected for diversity in age, sex, location, education (community) and profession (health knowledge experts). A series of eight focus groups were planned. From May 2023 to August 2023, seven online focus groups were undertaken separately with each cohort, to explore perceptions related to the core functions, content and design features. Their insights were used to develop the digital platform. Following a testing period, a final focus group was held with each cohort (March 2024) to evaluate the digital platform further. Focus groups were recorded with participant consent. Recordings and transcripts, live chats and interactive polls from the focus groups were analysed using inductive and deductive thematic approaches, with themes mapped to the Framework for the Design and Evaluation of MOOCs.

**Results:**

Twelve community members and ten health knowledge experts participated in at least one of the eight focus groups, with overall 86% attending five or more. Although some diversity existed in group opinions about the delivery and content, all participants emphasised the importance of using simple, easy‐to‐understand language and layout throughout, with the inclusion of a variety of statistics, personal stories and expert information. Focusing on emotional motivation was perceived as essential for engagement with the digital platform. Furthermore, being able to personalise the content and provide options for people to explore more advanced information (via external resources and a project‐specific website with trusted links) was considered advantageous.

**Conclusion:**

Co‐design with community and knowledge expert cohorts informed and enriched the development of the Love Your Brain digital platform. The co‐designed platform is currently being piloted in a feasibility trial.

**Patient or Public Contribution:**

People with lived experience of stroke, along with family/caregivers and members of the public, actively participated in the co‐design focus groups. The Love Your Brain Management Committee comprises lived experience stroke survivors and carers who worked in partnership with researchers and clinicians to provide oversight and guidance to the development and implementation of all stages of the study, including the preparation of this manuscript.

## 1 Introduction

Globally, stroke is a leading cause of death and disability [1], with one in four people estimated to experience a stroke in their lifetime [2]. Almost 90% of strokes globally are attributed to modifiable risk factors, such as high blood pressure, physical inactivity, elevated cholesterol, unhealthy diet, being overweight and smoking [3]. Effective control of these risk factors can prevent stroke, while also reducing the economic burden of cardiovascular disease (CVD) on the healthcare system [4].

Current strategies for stroke prevention primarily focus on pharmacological interventions to control risk factors and behavioural strategies to promote healthier lifestyles [5]. International guidelines play a vital role in guiding primary prevention strategies for stroke [6], with the Stroke Foundation's Living Clinical Guidelines [7] and the Australian CVD Risk guidelines [8] widely applied in Australia. Despite these efforts, the global burden of stroke continues to rise [1], highlighting the insufficient reach and efficacy of existing strategies. To address this, new approaches are needed [4, 9]. Broader consideration of government policies, along with socioeconomic, cultural and economic factors, is essential [4, 5]. Additionally, raising individual awareness about stroke remains a critical component of primary prevention [10].

There is an increasing interest in the use of digital technologies in preventative healthcare, with potential benefits in improving the translation of health information to the community [11, 12]. These technologies include smartphones and tablets (mobile health, mHealth), websites/digital platforms, wearable devices and short message services (SMS). Digital health interventions offer accessibility, convenience and the ability to personalise care delivery to promote healthy behaviours, provide education, support self‐management and facilitate behaviour change [13].

The benefits of digital health interventions for primary prevention education about stroke and other CVDs have been highlighted in several systematic reviews. Feigin et al. reported on the acceptability, feasibility and efficacy of various digital technologies and tools for managing stroke risk factors, including blood pressure control, smoking cessation, weight management and physical activity [10]. Other internet‐based, telemedicine and SMS interventions have shown benefits in managing selected CVD risk factors [14, 15, 16, 17]. However, the heterogeneity of digital health interventions, variations in the quality of reported studies and non‐adherence reported within the included studies contribute to variations in the effects across interventions [10].

Limitations of prior digital health interventions may be overcome through an appropriate study design. Poor adherence to digital health interventions can be addressed using a co‐design approach, where the needs, preferences and perspectives of users shape the final intervention or project [18, 19]. Involving stakeholders in this way shares power and decision‐making and can enhance the likelihood of successful development and user acceptance of the final product [20]. These principles were embedded into the Love Your Brain project, an initiative to co‐design and evaluate a digital health platform, comprising a Massive Open Online Course (MOOC) and a text messaging system, to empower Australians to manage their risk factors for stroke. The aims of the Love Your Brain digital platform are to provide education and improve retention of knowledge on stroke risk factors, increase the number of visits to general practitioners for an assessment of stroke risk factors and increase the uptake of healthy behaviours (e.g., adherence to prescribed medication). Co‐design underpinned the overall Love Your Brain project, with partnerships between people with lived experience (stroke survivors, carers and family) and researchers integral to informing and shaping the entire project. This paper specifically reports on the development of the digital platform where we aimed to apply co‐design principles to identify and refine the platform's core functions, content and design features to align with the needs and preferences of participants.

## 2 Methods

### 2.1 Study Design and Participants

The co‐design process was a participatory action research approach involving a series of focus groups that emphasised collaboration and action between stakeholders [21]. The process was iterative in that discussions from each focus group informed the content of subsequent focus groups. Our co‐design process involved two separate stakeholder cohorts: health knowledge experts, who could be health education or health promotion professionals (e.g., neurologist, general practitioner, nurse, allied health clinician and researcher), and adult community members (with or without a history of stroke).

### 2.2 Participant Recruitment and Focus Group Methods

Initially, participants were engaged through snowballing methods from April to May 2023, including via an email advertisement. This was circulated via study investigators to existing national clinical and research networks, community reference groups, consumer councils and newsletters (e.g., Stroke Foundation and Australian Stroke Clinical Registry). Interested participants (*n* = 16 health knowledge experts and *n* = 28 community members) were then purposively recruited considering diversity in sex, age, location (state), level of education (community members) and profession (health knowledge experts) (Table S1).

The online focus groups were facilitated by experienced qualitative researchers not involved with the development/preliminary work (T.P., female, Research Fellow, PhD; S.B., male, Research Fellow, PhD) and recorded with consent. Focus groups ran for approximately 60 min, with each cohort conducted in tandem (i.e., the same content for discussion was run with each cohort separately). Interactive polls, chat functionality and live summaries were included in all sessions. Other project team staff acted as observers, taking additional notes and assisting with technology as required. Data collection included videoconferencing recordings and transcripts, in addition to poll results and chat comments.

### 2.3 Focus Group Content

**Figure 1:**
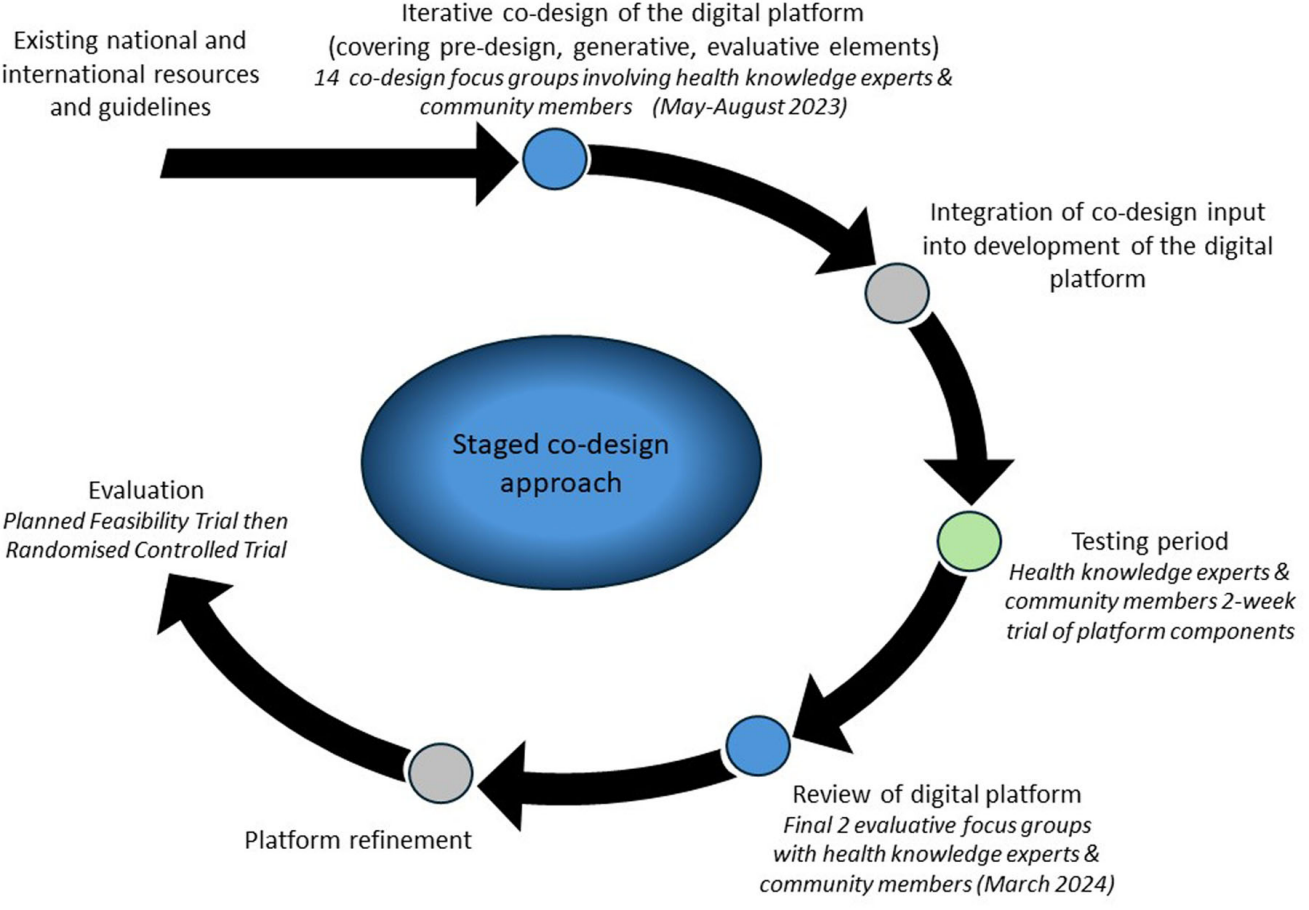
Co‐design approach of the Love Your Brain digital platform.

The overall structure and flow of the sequence of focus groups followed a staged co‐design process [22] including: learning about participant thoughts and experiences (pre‐design phase); generating new ideas related to the function, content and design features of the platform (generative phase); and obtaining feedback on these specific aspects (evaluative phase). The information from the initial 14 focus groups (i.e., seven with each cohort) was used to inform and help develop the digital health platform over 7 months. Participants then had the opportunity to test the digital health platform over 2 weeks, before the last evaluative focus group where feedback on the platform was sought from participants (Figure 1).

The research team had outlined a preliminary list of potential discussion topics to be covered within the focus groups based on their experience with prior work in primary stroke prevention. However, through the iterative collaborative process, discussions with participants directed what was important to consider in the digital platform and therefore informed the topics and order/flow of subsequent focus groups (Table 1). The first seven co‐design focus groups were held with each cohort (14 in total) over a 3‐month period (May–August 2023). A final focus group was undertaken with each cohort in March 2024, after the development of the platform and a 2‐week testing period for participants.

**Table 1:** Co‐design focus group content.

Focus group topics & methods	Design phases*, objectives and prompt questions
1. Introduction, purpose, important areas of inclusion	*Pre‐design: to understand participants' experiences and perceptions related to primary stroke prevention*
	Objectives Introduce the project, research staff and participants including ice‐breaking activities Identify what the community would like to learn about preventing stroke Determine what would make a stroke prevention program useful Discuss topics the digital platform should cover Prompt questions What do you think community members want to learn about preventing stroke? What would make a program about preventing stroke useful to the community? Which topics are most important to cover?
2. Stroke can happen to anyone (voting polls)	*Generative & evaluative: discuss & review ideas and rate format/functionality options*
	Objectives Summary from prior focus group +/− questions Identify stroke statistics that would motivate someone to participate in the digital platform. Determine the preferred format and presentation of stroke statistics (e.g. questions or statements, global or Australian, general or age/sex specific, different impact types, technical terms if explained, imagery) Prompt questions Why would someone want to participate in the digital platform? What numbers are important when talking about the impact of stroke?
3. What do people in the community need to know? (compiled videos, voting polls)	*Generative & evaluative: discuss & review ideas and rate delivery options*
	Objectives Summary from prior focus group +/− questions Identify the aspects of infographics and videos that effectively convey information about stroke (What is stroke? What is the impact of stroke? What are the signs of stroke [FAST]?) Prompt questions What format and features of the presentation style and delivery are preferred/not preferred? Consider: – Type of information (e.g. analogies), – Level of detail (e.g. physiology, anatomy) – Format type (e.g. infographic vs illustration) – Colouring – Text (e.g. written and spoken) – Credibility of the information – Video (length, background)
4. Stroke risk factors (compiled videos, voting polls)	*Generative & evaluative: discuss & review ideas and rate delivery options*
	Objectives Summary from prior focus group +/− questions Prioritise common and uncommon risk factors to include in the digital platform Review the style of messaging and level of detail (using blood pressure as an example) Prompt questions What are the important risk factors to be included in a program about preventing stroke? What information should be included for each risk factor? For example definition, effects, management. Case example of blood pressure used.
5. Taking action to prevent stroke (compiled videos)	*Generative: review and brainstorm ideas*
	Objectives Summary from prior focus group +/− questions Identify practical tips for making the most of a doctor visit for stroke prevention (getting people to the doctor, making the most of their visit, the most important question to ask, feedback on the Heart Health Check video) The next steps for enacting change to prevent stroke Prompt questions What would help people get to the doctor? What actions can people take to get the most of out of their doctor's visit? What is the most important question to ask you doctor about preventing stroke? What are the next steps to action to prevent stroke?
6. Delivery of the digital platform	*Generative: discuss & review ideas*
	Objectives Summary from prior focus group +/− questions Identify factors which may increase participant engagement with the digital platform (with the MOOC modules and text messages discussed separately) Prompt questions What factors may influence engagement with the MOOC? What factors may influence finishing the MOOC? What factors may influence people reading the text messages?
7. Text Message System (voting polls)	*Generative & evaluative: discuss & review ideas and rate delivery options*
	Objectives Summary from prior focus group +/− questions Identify factors which may increase participant engagement with the text messages, related to formatting and delivery (including the inclusion of the project name, the participant's name, links, phone number format, images, two messages as a bundle) Prompt questions What text message format would be preferable? – How messages are addressed – Inclusion of trusted links to websites – Message length – Use of images What aspects should be considered with the delivery of the text messages?
8. Review of the digital platform	*Evaluative: explore experiences of the co‐design process, plus feedback from testing the digital platform*
	Objectives Discuss feedback related the MOOC, text messages and co‐design process Prompt questions What was your experience with the MOOC/text messages (incl difficulties)? Did the process of co‐design meet your expectations? Is input from the process reflected in the digital platform?

FAST – face, arms, speech, time; MOOC – Massive Open Online Course; *based on the co‐design phases (pre‐design, generative and evaluative) defined by Sanders & Stappers [22].

Existing national and international resources and clinical guidelines for preventing stroke (e.g., guidelines produced by Stroke Foundation, World Stroke Organization and American Heart Association) initially informed the specific subject matter for discussion in the focus groups. Relevant segments from online videos and infographics were curated by the project team, extracted and compiled into videos for comparison and discussion during the focus groups on specific topics. Participants were provided with a 1–2‐page document before each focus group, outlining the topic and questions for discussion and including links to relevant videos. This document provided participants with an opportunity to familiarise themselves with the content for discussion at the next focus group.

All focus groups started with an Acknowledgement of Country [23] and an acknowledgement of people with lived experience of stroke. This was followed by terms of engagement in the co‐design process [24], to ensure participants were respected, supported and comfortable to contribute during the discussions. The first focus group for each cohort included an introduction of the facilitators and the wider project team, as well as a meet and greet for the participants. In addition, the overarching objectives and components of the Love Your Brain digital platform (MOOC and text message system) were presented. This also included a discussion to explore participants' experiences and perceptions related to primary stroke prevention, which informed subsequent focus group content.

The following focus groups (2–7) started with a brief recap of the previous group discussion, followed by an introduction to the current topic. These sessions included a review of relevant videos and graphics, facilitated discussions using prompt questions and concluded with a summary provided by the facilitator (Table 1). In general, participant views on content (topic importance, usefulness, type of information and level of detail), features and formatting (phrasing, visualisation and terminology), and functionality of each component and the digital program (in terms of frequency and length) were explored. For the first seven focus groups, participants from each group were also provided an option to complete an online evaluation survey to provide additional free‐text information related to the topics discussed.

The final eighth (evaluative) focus group with each cohort was to obtain participants' experiences and feedback related to the testing of the MOOC and text message system. This focus group also included a wider discussion about participants' involvement in the co‐design process for developing the platform (reported elsewhere). Participants were also advised that they could provide direct feedback to the study team via email at this stage. Throughout the iterative process, participants were active partners in the research, contributing insights and experiences. The collaboration with the research team was integral in shaping decisions around what was important to consider and include in the digital platform, as well as the design and functionality.

### 2.4 Analysis

Videoconferencing recordings and transcripts, comments in the ‘chat’, live summaries, in‐depth notes from the facilitator and observers taken throughout each focus group, and any additional free‐text responses provided in surveys/emails were entered into Microsoft Excel to organise and manage the data for thematic analysis [25]. A combination of both inductive and deductive approaches to coding was undertaken [26]. This allowed for the flexibility to discover new themes grounded in participants' experiences, while also exploring specific themes of interest related to the platform (e.g., mode of delivery and presentation modalities) [27].

The initial coding was conducted by R.F. Emergent themes and sub‐themes were independently reviewed by T.P. and V.S. and discussed more widely within the research team to ensure that interpretation and meaning were maintained. To assist in conceptualising the needs and preferences of the participants, themes and sub‐themes were mapped to the dimensions of the Framework for the Design and Evaluation of MOOCs described by Grover et al. [28]. While the focus of our study extended more widely than MOOCs, we felt the dimensions of this framework applied to our overall digital health platform. Specific dimensions considered included: (i) the learner background (why people would engage with the program); (ii) interactive environment (i.e., the content, instruction, assessment and target audience); (iii) technology and infrastructure (accessibility and inclusivity) and (iv) evidence‐based improvement (areas to improve the learning environment). Resultant themes and sub‐themes from the analysis shaped the content and design of the digital platform, with important elements being incorporated into the prototype subsequently tested by participants (Figure 1). Illustrative quotes from participants have been provided to reflect each theme.

Ethical approval for this study was received from the Monash University Human Research and Ethics Committee (#35899). Universal Trial Number U1111‐1305‐2964. All focus group participants provided written or verbal consent. This study has been reported according to the Consolidated Criteria for Reporting Qualitative Research (COREQ) checklist (Supporting Information) [29].

## 3 Results

A total of eight focus groups were conducted with the health knowledge experts (*n* = 10, all attended at least one focus group, 80% attended five or more; 60% > 35 years, 80% female) and eight focus groups with community members (*n* = 12 attended at least one focus group, 92% attended five or more, 58% < 65 years, 58% female, 11 with lived experience of stroke as a survivor or carer) (Table S1). The health knowledge experts included five researchers and five clinicians (a nurse, physiotherapist, occupational therapist and two speech pathologists).

The iterative co‐design process was instrumental in shaping the content and flow of the planned focus groups. Important examples include the addition of a targeted focus group dedicated to specifics of ‘*Taking Action*’ (focus group 5) and allocation of dedicated time to explore participant views on misconceptions and important ‘uncommon’ risk factors (focus group 4).

A summary of the themes and sub‐themes arising, linked to the Design and Evaluation dimensions are provided in Table 2. Additional participant quotes are provided in Table S2.

**Table 2:** Themes, sub‐themes and examples from the co‐design process mapped to the Framework for the Design and Evaluation of Massive Open Online Courses.

Design and evaluation dimensions*	Themes and sub‐themes	Example participant quotes	Suggested inclusions in the digital health platform
Learner background and intentions (why people engage with the program)	Motivation		
	It won't happen to me (stroke is an issue at all ages)	‘*We've got a 16‐year‐old in our acute stroke ward at the moment … it can happen to really young people as well’* (Health Expert, ID_07)	Provide statistics and stories to emphasise that stroke is an issue at all ages
	Impact of stroke	‘*… it [stroke, affects] everything. It basically affects people in every second of every day of their life in some way or another*’ (Community, ID_12)	Highlight the range of physical, cognitive, emotional and social effects on the individual and wider family and support network
		‘*I've returned to work, but I'd sort of estimate I'm doing only about 40% of what I was*’ (Community, ID_19)	Include information related to survival, disability and other tangible functional examples of impacts
	Stroke is preventable	‘*Strokes are preventable, 80% of strokes are preventable. To know that we are in power to do things, to change that, and to be one of the 80%’* (Community, ID_13)	Statistics to emphasise that stroke is preventable, and simple changes can make a difference to the risk of stroke, including personal stories
	Secondary benefits	‘*As a young person myself who thinks I am healthy, and I won't have a risk for stroke, an incentive for me to engage with this platform could be educating my family and other people who may have high blood pressure so that they can manage their risk for stroke*’ (Community, ID_17)	
	**Empowerment and agency**		
	Knowledge is power	‘*Prevention is so much about empowerment and agency of people to ask questions and to advocate for themselves, particularly around their health*’ (Community, ID_13)	Consistent messaging that there does not need to be a problem to seek medical advice—prevention is important
			Consider language related to agency—‘things you can control vs modifiable’
	Ability to make choices and change	‘*You're the bus driver of your life, but there are people on the bus that can help guide, support, encourage the driver’* (Health Expert, ID_08)	Provide questions people can ask to advocate for their health and how to approach their health provider
		*‘There is an element of control of what they do now that can have an impact later’* (Health Expert, ID_08)	There is a clear link between risk factors and how these lead to stroke
			Acknowledge that change is difficult—it might take more than one attempt to enact change
		‘	Include recommendations about who can assist in making changes
			Enable the choice of risk factors individuals may want to learn more about or are relevant to them
Interactive environment (related to the specific content, instruction, assessment and target audience)	Presentation modalities		
	Simplicity in presentation	‘*I think it needs to be easy to use. Some people I find are surprisingly lazy, and if it's not easy to use the first time, they just won't go back and try again’* (Health Expert, ID_07)	Follow health literacy principles—lay language, bullet points, large font, no big blocks of text and use images
			Consistency in how messages are delivered in the same format
			Minimise background music in videos
	Communication style	‘*There are benefits of presenting it [information] in multiple modalities, particularly when people are trying to absorb new information*’ (Health Expert, ID_08)	Include multiple modalities throughout both MOOC and text messages as much as possible
	Functionality considerations	‘*Having a “progress bar” at the top is helpful*… *if you don't have that [you're] 50% of the way through, you can feel a little bit lost*’ (Health Expert, ID_02)	Building trust is important through the use of a consistent phone number for messages, signed off from ‘The Love Your Brain Team’
			Include a progress bar in MOOC modules
			Incentives for completion—gamification or certificates
	Content		
	Accuracy of information	‘*To a certain extent it [getting cholesterol checked] seems simple but misleading, because my understanding is it involves fasting, then testing your blood, then it goes to pathology, then it comes back … it's not a 20 minute exercise*’ (Community, ID_21)	Figures and information presented should be the most recent and accurate
	Ease of understanding	‘*Don't make any assumptions about peoples understanding [of physiology],… taking a step back and showing that these vessels go through at your body and what they do… I thought this was a good place to start’ (Health Expert, ID_06)*	Avoid technical terms if possible; include a glossary if any technical terms used
			Explain all concepts in full
			Utilise pre‐existing resources or systems
	Important topics of focus	Why risk factors are important, including misconceptions	Blood pressure and atrial fibrillation are essential risk factors to focus on
		What to look for in monitoring risk	Include practical examples of simple and achievable strategies and actions to achieve change
		How to apply knowledge	Emphasise key take‐home messages
	Personalisation/tailoring		
	Relatable information	‘*1 in 4 people are going to have a stroke. What is that really like? How does that compare to something that maybe they're familiar with, maybe breast cancer?*’ (Health Expert, ID_08)	Include information that is personable and relatable—personal stories and statistics compared to more common diseases
		‘*I really think that to capture young people, you're going to need video messages from a young person saying I didn't think this could happen to me, but it did, so, please, get yourself checked*’ (Community, ID_20)	
	Experience to suit personal needs	‘*Some modules…, once you've opened it, you've got to keep going. But if you're able to just pause, and it will keep going where you left off when you're able to get back to it ‐ I thought that would be helpful, too*’ (Health Expert, ID_02)	Ability to personalise experience, for example, text messages—only related to relevant risk factors chosen, or MOOC—include core modules and information, with option for more detail if interested
		‘*Some people are happy with the basics. Some people are thirsty for more, so then you could tailor the content… maybe some basics and then advanced level, so people can choose how much they take in.*’ (Health Expert, ID_02)	For MOOC, being able to ‘stop’ and ‘start’ and be self‐paced
			‘Opt‐out’ opportunity for text messages
			Reminders and prompts to come back and finish modules (MOOC), or to respond to text messages
			Address all text messages to participants by name
	Opportunities for wider learning		
	Generation of own knowledge	‘*[in MOOCs I have done] there were links where you could just go to the web and download or investigate particular links, they were all there…I liked it…’* (Community, ID_21)	Access to additional resources for wider learning or support, including links to trusted websites and a project‐specific website with resources for text messages
	Social connectivity and self‐reflection	‘*Maybe having a chat/discussion forum to ask questions or talk to other participants? That's then an extra active component of the intervention*’ (Health Expert, ID_06)	Offer a chat discussion with other MOOC users or a way to respond to messages
			Include activities where people ‘commit to change’
Technology infrastructure (accessibility and inclusivity)	Accessibility		
	Compatibility with different technologies and devices	‘*My phone was $200, and it's not all the time connected [to internet]. So I can't watch videos and things without going through quite a process which is sometimes too much in a public place. I'm probably lagging behind the curve with technology*.’ (Community, ID_14)	Accessible on different devices—tablets, smartphones and laptops
			Have technical support available
	Inclusivity		
	Ability to cater to the learning needs of diverse populations	‘*[with] anything over 3 syllables there is a high risk that people won't understand what you're talking about*’ (Health Expert, ID_06)	Include stories/pictures/information from diverse populations
		‘*Having the ability to put the captions on with the videos, as a person who cares for someone with aphasia, captions are good*’ (Community, ID_20)	Consideration of the complexity of language
			Include transcripts and captions with videos
			Minimise background music, having multiple people speaking concurrently
			Consider the colours used in diagrams
			Available in different languages
Evidence‐based improvement (ongoing assessment and improvements to the learning environment)	Ongoing improvement and testing	‘*It was a lot easier to watch and listen [to videos] in a more natural setting, the ones where the presenter was superimposed on a background I found a little bit distracting*’ (Community, ID_19)	Review technical feedback
		‘*…the contrast [in images] may present problems for the visually impaired, it is certainly not approachable for me, it is too dark and shaded’* (Community, ID_21)	Additional focus on diversity—include cultural‐specific statistics, have information available in different languages or links to websites where information can be sourced in different languages, additional bolding of key words in text, review all image colour/contrast for vision impaired

Abbreviation: MOOC, Massive Open Online Course.

* Adapted from Grover et al. 2013—Framework for the Design and Evaluation of MOOCs [28].

### 3.1 Learner Background and Intentions (Why People Engage With the Program)

#### 3.1.1 Motivation

The consensus from participants of both groups was that individuals often underestimate their risk of having a stroke, assuming they are immune because of their perceived health or age.

‘*There is this natural apathy. I was the same. Strokes won't happen to me. I'm fit, healthy, doing all the right things’*

Community, ID_15

Therefore, to engage individuals in a primary prevention program for stroke, the importance of ‘*capturing attention*’ was emphasised through the use of statistics and personal stories to highlight that ‘*stroke can happen at any age … [it can] happen to men, women, and children*’ and, is, therefore, relevant to everyone. There were varied preferences reported between groups about the use of national or global stroke statistics, but there was a universal perception that outlining the impact of stroke to ‘*motivate people emotionally*’ was a useful means of engagement. Emphasising the impact of stroke on individuals was considered essential, particularly for ‘invisible disabilities’ such as difficulties with producing and/or understanding language (i.e., aphasia) and persistent feelings of cognitive and physical tiredness, weariness or low energy (i.e., fatigue). Additionally, recognising the broader impacts and burden on families was seen as crucial. Participants also described the importance of emphasising that most strokes are preventable, and people understanding their own risk and making small changes to their health can make a difference in reducing the likelihood of stroke.

#### 3.1.2 Empowerment and Agency

Participants identified that empowerment and agency were crucial in any health prevention intervention. Understanding that ‘*knowledge is power*’ was a clear message, and the importance of individuals taking charge of their health by seeking advice, asking questions and advocating for themselves was commonly reported throughout the focus groups.

‘*Gathering information is power … but you are still the expert of you … you are the boss of your body…’*

Health Expert, ID_08

It was recommended that simple suggestions around being prepared and understanding ‘what questions to ask’ at health professional appointments be included in the digital platform. Ensuring the connection between risk factors and how these contribute to the development of stroke was perceived as crucial in enabling more informed decision‐making.

‘*I guess, I'm aware that blood pressure and cholesterol need to be kept within sort of certain bounds. But I never knew why those were important, and I certainly didn't associate them with stroke’*

Community, ID_19

Participants, both community and health knowledge experts alike, also stressed the importance of providing an understanding that everyone has a level of control over their lives. It was suggested that including stories of people who have reduced their stroke risk could encourage others to realise that small changes can make a difference.

‘*To know that we are in power to do things, to change [our risk of stroke]’*

Community, ID_13

Allowing individuals to choose specific topics they wanted to learn more about within Love Your Brain was another way to address the element of control. One suggestion was to have participants identify which risk factors are important and relevant to them, thereby guiding the potential changes they aim to achieve.

### 3.2 Interactive Environment (Related to the Specific Content, Instruction, Assessment and Target Audience)

#### 3.2.1 Presentation Modalities

Ensuring that the information presented throughout the digital platform is visually appealing, with simplicity in the presentation, was perceived to be fundamental by all. Adhering to health literacy principles, such as using lay language, larger font, bullet points and ‘jargon‐free’ explanations, was recommended for both the MOOC and text messages to enhance comprehension and engagement. Where possible, the inclusion of simple diagrams and visuals with the use of colours was also recommended.

From the co‐design process, it was clear that the inclusion of multiple modalities would be beneficial to cater for individuals' diverse communication styles, learning preferences and cultural considerations. For the MOOC, a range of multimedia options including text, figures and informatics, short videos, animations and interactive tools such as knowledge tests or self‐assessment ratings were highlighted as useful to cater to various learning styles and preferences. It was perceived that input from subject matter experts would enhance the creditability of information provided within the digital platform. Including personal stories throughout was considered essential to foster a more effective connection. There were differing opinions about specific multimedia components (e.g., TikTok‐style video, hand drawings, animations and computer‐generated imagery). However, participants felt that minimising distractions such as background music in videos and image overlays while people were talking was important so as not to overwhelm individuals.

Modality elements related to text messages were limited. Participants varied in their preferences related to the inclusion of images within text messages. It was raised that wider consideration of individuals' data or device limitations may be required if images were to be sent in text messages. Additionally, images without context or with limited text were potentially felt to be confusing, especially if they were not personalised to the individual. Ultimately, participants agreed that sending images within text messages was not worthwhile, considering the additional cost per message, with little perceived benefit. Trustworthiness was also highlighted as important in relation to text messages. Suggestions such as having the text messages sent from a consistent phone number or email address and signed off from ‘The Love Your Brain Team’ were perceived to be important to help develop trust and ensure messages were easily recognisable.

Participants also felt that the inclusion of functional aspects within the digital platform, such as the use of a progress bar (MOOC) and incentives to complete the MOOC modules or other tasks encouraged via text messages, would significantly improve overall user engagement and motivation.

#### 3.2.2 Content

Another important theme related to the content of the material provided within the digital platform. Ensuring that the information provided was consistent, accurate and aligned with guideline recommendations was raised. Participants from both groups also reported that it was important to assume individuals will have no underlying knowledge related to stroke and risk factors. Therefore, explaining all concepts in full was essential, including a ‘glossary’ in the MOOC and project‐specific website where additional information could be found (text messages), and this was recommended. Clear and engaging communication, including avoiding technical jargon and using analogies and visual aids such as informatics, was also highlighted to enhance comprehension.

‘*Say someone has aphasia, having something [information] which has limited words, avoiding complex language and sort of focuses possibly more, on the graphics and the pictures.[something that is] easy on the eyes to look at as well’*

Health Expert, ID_07

Other reported areas to cover in the digital platform included ensuring there was a clear link between each risk factor and the relationship with stroke, addressing any misconceptions about risk factors. This included providing clear examples for how to monitor your risk. Including practical examples of simple and achievable strategies and actions to achieve behaviour change, including vital take‐home messages or ‘*action statements*’ throughout, was perceived as important to encourage individuals to change their behaviour.

Personalisation and tailoring were also emphasised as crucial aspects of effective health education for primary prevention. Providing information individuals can connect to, both through personal stories and relatable age‐ and sex‐specific statistics, was seen as a powerful tool to capture attention and raise awareness, particularly among young people. Additionally, participants expressed that tailoring the overall experience of all elements within the digital platform would significantly enhance user engagement and satisfaction. This included ensuring all text messages were personalised, such as being addressed to the participant by name, sent at the chosen time of day and consistent with the preferred message frequency. Allowing individuals to learn at their own pace and convenience by having the MOOC self‐paced, with the ability to stop and return as needed, was agreed upon by all. Participants from both groups valued the ability to choose which risk factors to target in their text messages, and similarly, had a choice in relevant risk factor modules in the MOOC. This personalisation was viewed as important in empowering individuals to choose the level of detail for their engagement as well as promoting active learning and knowledge retention.

#### 3.2.3 Opportunities for Wider Learning

Encouraging the continuation of education and knowledge generation throughout and beyond the program was perceived as important. This included leveraging opportunities for wider learning through access to existing resources and support services as part of the digital platform.

‘*…there's things [resources] around that you could sort of pull in that are already kind of available…you could just link people out to other resources or bring those elements in’*

Health Expert, ID_06

Inclusion of additional links to resources within the MOOC was considered straightforward. However, with the increase in phishing attacks and fraudulent websites, many participants reported they would be cautious in directly accessing links provided in text messages, particularly if message shorteners (e.g., bit.ly links) were used. Consequently, it was suggested that consideration should be given to other, more trustworthy means to provide access to these resources that aligned with the MOOC content for those receiving text messages. Participants perceived that the use of a webpage for the project, which links to further resources, would be a potential solution. Many participants also felt there was a possible benefit beyond the individual completing the digital platform, through sharing what had been learnt with family and friends.

Participants also reflected on the benefit of social connectivity for engagement and motivation to take part in prevention activities, with suggestions to incorporate ‘*chat’* forums for discussion with other MOOC users/administrators, or the ability to respond to text messages. Other features to encourage self‐reflection were also valued, with suggestions of interactive activities to ‘*measure your own stroke risk’*, including knowledge checks, and reflective prompts to encourage active participation, for example, ‘*When was the last time you had your blood pressure checked?’* or ‘*Do you understand why you are taking your medications?’*.

### 3.3 Technology Infrastructure

#### 3.3.1 Accessibility

Considering issues related to the accessibility of both the MOOC and text messages were raised by participants. Ensuring the digital platform was responsive and user‐friendly if accessed on different devices was felt to be essential in the overall success. For example, participants reported that the MOOC should be compatible with tablets, laptops or mobile phones without affecting the functionality, navigation or user experience. Messages should be accessible across various devices, regardless of screen sizes, data limitations and hardware. Having technical support available for troubleshooting was also recommended.

#### 3.3.2 Inclusivity

Participants voiced the need to ensure the information provided within both the MOOC and text messages was inclusive to allow users, regardless of their abilities, backgrounds or circumstances, to access and understand the content. Participants discussed the need to consider aphasia‐friendly formats, including ‘*font size and bolding*’, and terminology related to the level of education. ‘*Colour and contrast*’ were felt to be important for those with low vision, and providing transcripts for videos was deemed necessary for those who may be hearing impaired. Considering Australia's multicultural society, participants in both groups also raised the issue of having information available in different languages.

### 3.4 Evidence‐Based Improvement (Ongoing Assessment and Improvements to the Learning Environment)

For participants, being able to test the digital platform and then give feedback on their experience during the last focus group or via email was important in the evaluative phase of this co‐design process. Overall, the feedback was positive, highlighting that the MOOC and text message system were informative and concise. Very few technical issues were reported, and these issues were primarily centred around formatting challenges on different devices when accessing the MOOC. The solution for those receiving text messages to access a project‐specific webpage with links to external resources was felt to be successful in providing a trusted means to access additional information.

Suggested improvements related to providing additional clarity around certain terms and concepts, for example, defining a ‘standard drink’ or a ‘serve of fruit or vegetables’. Further emphasis was placed on inclusivity and accessibility throughout the platform. This feedback noted the importance of added diverse representation in videos and stories throughout, additional bolding of key words in the text, further review of colours, contrasts and backgrounds used in images and videos, as well as including links to existing resources and health information in other languages (e.g., Stroke Foundation of Australia https://strokefoundation.org.au/about-stroke/learn/languages). These suggestions will be incorporated into the digital platform before further evaluation in a feasibility trial and a randomised controlled trial.

## 4 Discussion

We describe the co‐design of a digital platform that includes a MOOC and text message system for primary stroke prevention, intended to enhance health knowledge and promote self‐management of risk factors. The iterative co‐design process included eight focus groups with community members and eight focus groups of health knowledge experts to understand the needs and ideas of the target population. Suggested functions, content and design features were discussed by participants, with elements being incorporated into an initial platform prototype that was subsequently tested by participants. Post‐testing feedback was used to refine the platform (evaluative phase), which will be tested within a feasibility trial as the next step, before a fully powered effectiveness randomised controlled trial. The iterative process enabled us to continually revise and evaluate the design and features of the digital platform to ensure it reflected the needs of the target population.

A recurring theme in the co‐design focus groups was the underlying motivation that would drive individuals to engage in the stroke prevention digital platform. Participants identified that capturing the attention of individuals was crucial for effective engagement in any primary prevention program. Consistent with prior literature [30], helping individuals relate to the issue by ensuring they recognise the relevance to their own lives was vital. The use of personal stories and relevant age‐ and sex‐specific statistics was suggested as important emotional motivators, which could be enhanced by the inclusion of more diverse representation. Also essential was to encourage an element of empowerment, with the understanding that individuals have control over their health and that small lifestyle changes can have a profound impact on preventing stroke.

Significant attention was also placed on how best to convey information to a broad audience within the digital platform. The effectiveness of communication strategies was a recurring theme. Simple, jargon‐free messaging and features like larger fonts and aphasia‐friendly formats to ensure inclusivity were considered important for use in both text messages and the MOOC. The importance of these strategies in enhancing the accessibility and effectiveness of health education materials is well highlighted in the literature [31], with comprehensive resources and guidance available [32, 33]. It was also suggested that the inclusion of the ‘glossary of terms’ in the MOOC and linking to the project‐specific website for those receiving text messages would cater to individuals who may want additional or more detailed information. These recommendations align with broader findings from health promotion research, which outline the need to balance using simple messaging that is comprehensive, but not too overwhelming or confusing [34]. During the co‐design process, variation in preferences for learning modalities was seen, emphasising the need for diverse formats to be included in the digital platform to engage a wide audience. This was not surprising considering that demographics, including education level, ethnicity, culture and health literacy, can play a significant role in information‐seeking behaviour, especially in health‐related contexts [35, 36]. Similarly, authors of a systematic review of digital interventions highlighted the need to move away from a ‘one‐size fits’ all approach and the importance of utilising a mix of modalities for delivering content [37]. Although the focus groups suggested that certain modalities such as figures, tables, informatics, short videos and animations were more applicable to the MOOC, they also considered that the format of text and interactive messages/tools was relevant to the text messages.

The importance of ensuring that digital technologies focused on self‐management or prevention are personalised, regardless of the specific purpose and audience, is widely recognised and well‐documented in healthcare literature [38, 39, 40, 41]. This personalisation, or tailoring of the experience to the individual user, was also strongly emphasised by participants in our focus groups as a crucial factor for enhancing engagement. The ability to customise the digital technology to meet individual needs is closely tied to its practical and social acceptability, which are also critical factors in ensuring ongoing use [38]. Within our digital platform, participant suggestions led to the inclusion of core modules in the MOOC and text messages about stroke generally, with additional modules (MOOC) and messages (text) related to identified risk factors and areas for improvement being available for each individual based on their needs. Ensuring that text messages in the digital platform are addressed directly to participants and sent at the chosen time of day, along with offering self‐paced completion of the MOOC, were also recognised as essential for fostering a personalised user experience.

The growing burden of stroke provides evidence that the current prevention measures for primary stroke and CVD are not effective or used widely enough [4]. In this study, we addressed the need for more far‐reaching, accessible, lower‐cost and targeted primary prevention strategies, particularly for individuals at risk of stroke [40, 42]. While the majority of participant suggestions from the co‐design process were incorporated into the final design of the MOOC and text message system, several barriers limited the inclusion of all suggestions. For example, some personalisation recommendations, such as having text messages sent at days and times specified by the user, were not possible due to technical limitations. However, days/times deemed most preferable by other mHealth interventions were chosen [43]. Due to character count limitations, it was also not feasible to sign off every text message from ‘The Love Your Brain Team’. Instead, priority was given to delivering essential stroke‐related information, as these health messages were the focus. Since all messages originated from the same phone number and would appear within the same message thread on users' devices, this consistency was deemed sufficient for users to identify the sender easily. Although the importance of addressing the needs of linguistically diverse groups was recognised, current limitations of the project meant that the MOOC and text messages have been developed only in English, with links to resources in multiple languages. After feasibility and effectiveness testing, we recognise the importance of ensuring the information is available in multiple languages. Additionally, the recommendation of a chat function was incorporated into the MOOC through an online discussion board. This is accessible during the final module, noting that monitoring is required with staff to provide ongoing resources and support. In the future, advancing technologies with generative AI may further enhance personalisation by enabling real‐time, adaptive interactions that can more closely align with each individuals' learning preferences [44].

The present study has several strengths and limitations. The co‐design approach, with active collaboration and shared decision‐making between participants and researchers, ensured the resultant digital platform was user‐centred and effective in addressing the needs of the target population. This was informed by the iterative co‐design focus groups but also influenced by wider collaborations and partnerships with people with lived experience and researchers on the project management committee (explored further in a separate publication), who were integral in guiding the project and wider decision‐making. Conducting separate focus groups for the health knowledge experts and community members ensured an equal voice for both health knowledge experts and community members, mitigating any power imbalance and fostering cohesion within the separate cohorts. However, this may have limited any potential mutual learning between participants from each cohort [45]. Although participant feedback related to the co‐design process was positive [46], further collaborative input could have been enhanced by the use of more formal consensus‐building techniques or additional capacity‐building sessions.

The commitment required for eight focus groups may have also created a barrier to recruitment, limiting our sample size. Nevertheless, the iterative approach, inclusion of diverse participants, structured facilitation and generation of actionable insights to address the function, content and design features ensured adequate information power [47]. Including both health professionals and researchers involved in education or health promotion, as well as community members, enhanced the platform's relevance and applicability. Most community members were people with lived experience of stroke or carers, three with experience of having, or caring for a person with, aphasia. All were involved with the Stroke Foundation StrokeSafe program [48]. This program educates the community about stroke, so this cohort had an intimate knowledge of community expectations and needs related to stroke prevention. While we acknowledge that further expanding recruitment specifically to community members with no history of stroke would have diversified perspectives, project time frames limited this option. Further user input from these community members will be sought in Stage 2 testing of the digital platform, which includes an evaluation component to address these limitations and further refine the platform. We acknowledge that our co‐design process did not incorporate the viewpoints of various culturally and linguistically diverse (CALD) communities, including Aboriginal and Torres Strait Islander communities. Following the recommendation to ensure that the digital platform is personalised, we plan to extend this project to conduct further co‐design with specific CALD communities after the effectiveness of the platform within the general community is evaluated. The use of the Framework for the Design and Evaluation of MOOCs added depth to the interpretation of results, lending to a more informed understanding of the needs and preferences of participants related to the core content, design and features to be included in the digital platform. Additionally, conducting the focus groups online also allowed wider involvement with reduced time burden for participants while still including interactive components to increase engagement with chat functionality and polls.

Our study contributes further evidence to the growing body of literature on the benefits of co‐design, particularly in developing digital technologies [41, 49, 50]. A separate publication has been dedicated to the recruitment, coordination, participant engagement and satisfaction of the co‐design process for the Love Your Brain study [46]. While specific to the Australian healthcare context and directly focused on primary prevention of stroke, many of our findings are generalisable to primary prevention of chronic disease, as well as findings relevant to health promotion and prevention. The applicability of our findings extends beyond MOOCs and text messages to a broader use of digital technologies.

## 5 Conclusion

This study provides important details related to the function, content and features of a stroke prevention digital platform designed for the community. It contributes to the growing body of research on co‐designing digital health interventions for disease prevention. By leveraging the combined expertise of healthcare professionals and the lived experience of community members, the Love Your Brain digital platform has the potential to be a more effective and user‐centred tool for improving stroke prevention knowledge and reducing stroke risk in Australia.

## Author Contributions

**Tara Purvis:** methodology, data curation, formal analysis, writing – original draft, writing – review and editing, funding acquisition. **Catherine Burns:** data curation, writing – review and editing, project administration. **Seamus Barker:** data curation, writing – review and editing. **Monique F. Kilkenny:** conceptualization, methodology, writing – review and editing, funding acquisition. **Seana L. Gall:** conceptualization, methodology, funding acquisition, writing – review and editing. **Christine Farmer:** writing – review and editing, Project administration. **Vaishnavi Sudhakar:** formal analysis, writing – review and editing. **Dominique A. Cadilhac:** funding acquisition, writing – review and editing. **Brenda Booth:** funding acquisition, writing – review and editing. **Janet E. Bray:** funding acquisition, writing – review and editing. **Jan Cameron:** funding acquisition, writing – review and editing. **Lachlan L. Dalli:** writing – review and editing. **Stephanie Ho:** writing – review and editing. **Eleanor Horton:** funding acquisition, writing – review and editing. **Timothy Kleinig:** funding acquisition, writing – review and editing. **Lisa Murphy:** funding acquisition, writing – review and editing. **Mark R. Nelson:** funding acquisition, writing – review and editing. **Muideen T. Olaiya:** funding acquisition, Writing – review and editing. **Amanda G. Thrift:** funding acquisition, Writing – review and editing. **Rosanne Freak‐Poli:** conceptualization, methodology, data curation, formal analysis, project administration, writing – review and editing.

## Ethics Statement

Ethical approval for this study was received from the Monash University Human Research and Ethics Committee (#35899).

## Conflicts of Interest

M.F.K. and D.A.C. are members of the Australian Stroke Clinical Registry Management Committee; A.G.T. is a previous Board Member of Stroke Foundation; M.F.K. is a member of the Research Advisory Committee of Stroke Foundation; S.L.G. is Chair of Stroke Foundation's Stroke Prevention Advisory Committee and a member of their Clinical Council. M.R.N. reports membership of a Novartis lipids advisory board outside the submitted work. All other authors have no conflicts of interest to declare.

## Supporting information

Response Supplemental Material Final clean.

## Data Availability

Data are not available as participants were informed and consented to only non‐identifiable excerpts of their data being used in publications, and they did not consent to data sharing.
