# Supplementary material for: Co‐Designing a Digital Stroke Prevention Platform: Leveraging Lived Experience and Expert Advice
Source: Health Expect. 2025 May 22;28(3):e70293. doi: 10.1111/hex.70293 (PMC12098301; doi:10.1111/hex.70293)
Supplement: Supplementary file 1 — Response Supplemental Material Final clean. [file HEX-28-e70293-s001.docx]

**Co-designing a digital stroke prevention platform: Leveraging lived experience and expert advice**

Tara Purvis^1^, Catherine Burns^1^, Seamus Barker^2^, Monique F. Kilkenny^1,3^, Seana L. Gall^2^, Christine Farmer^2^, Vaishnavi Sudhakar^1^, Dominique A. Cadilhac^1,3^, Brenda Booth^4^, Janet E. Bray^5^, Jan Cameron^1^, Lachlan L. Dalli^1^, Stephanie Ho^4^, Eleanor Horton^4^, Timothy Kleinig^6,7^, Lisa Murphy^4^, Mark R. Nelson^2^, Muideen T. Olaiya^1^, Amanda G. Thrift^1^, Rosanne Freak‐Poli^1,5^

^1^Stroke and Ageing Research, Department of Medicine, School of Clinical Sciences at Monash Health, Monash University Clayton, Melbourne, Australia

^2^Menzies Institute for Medical Research, University of Tasmania, Hobart, Australia

^3^Stroke Theme, Florey Institute of Neuroscience and Mental Health, University of Melbourne, Melbourne, Australia

^4^Stroke Foundation, Melbourne, Australia

^5^School of Public Health and Preventive Medicine, Monash University, Melbourne, Australia

^6^Department of Neurology, Royal Adelaide Hospital, Adelaide, Australia

^7^Department of Medicine, University of Adelaide, Adelaide, Australia

**Online Supplemental Inclusions:**

Supplemental Table 1: Characteristics of the Health Knowledge Experts and Community Members involved with the co-design process

Supplemental Table 2: Summary of additional quotes related to the derived themes and sub-themes

COREQ (COnsolidated criteria for REporting Qualitative research) Checklist

**Supplemental Table 1: Characteristics of the Health Knowledge Experts and Community Members involved with the co-design process**

|  | **Health Knowledge Experts**  **N=10, n (%)** | **Community Members**  **N=12, n (%)** |
| --- | --- | --- |
| **Age** |  |  |
| <35 years | 4 (40) | 1 (8) |
| 35-54 years | 4 (40) | 4 (33) |
| 55-74 years | 2 (20) | 7 (58) |
| 75+ years | 0 (0) | 0 (0) |
| **Sex** |  |  |
| Woman | 8 (80) | 7 (58) |
| **Location (State)*** |  |  |
| New South Wales | 6 (60) | 3 (25) |
| Victoria | 4 (40) | 2 (17) |
| Queensland | 0 (0) | 2 (17) |
| Western Australia | 0 (0) | 0 (0) |
| South Australia | 0 (0) | 1 (8) |
| Tasmania | 0 (0) | 3 (25) |
| Australian Capital Territory | 0 (0) | 1 (8) |
| Northern Territory | 0 (0) | 0 (0) |
| **Highest level of education** |  |  |
| Less than Year 12 equivalent | 0 (0) | 0 (0) |
| Year 12 equivalent | 0 (0) | 0 (0) |
| Certificate/Diploma | 0 (0) | 3 (25) |
| Bachelor Degree | 2 (20) | 6 (50) |
| Postgraduate Degree | 8 (80) | 2 (25) |
| **Professional Expertise** |  |  |
| **Clinician** | 5 (50) | - |
| Medical | 0 (0) | - |
| Nurse | 1 (10) | - |
| Allied Health^ | 4 (40) | - |
| **Researcher** | 5 (50) | - |

*in Australia, ^including physiotherapists, occupational therapists, speech pathologists

**Supplemental Table 2: Summary of additional quotes related to the derived themes and sub-themes**

| **Design & Evaluation dimensions*** | **Themes and sub-themes** | **Example participant quotes** |
| --- | --- | --- |
| ***Learner background & intentions*** *(why people engage with the program)* | **Motivation**   - It won’t happen to me (stroke is an issue at all ages) | “*When it happened to my daughter it was completely unexpected. When it happened to my elderly dad, that's not so unexpected at his age”* (Community, ID_20)  *“I think, having a stroke statistics, whatever that might look like first, is almost kind of capturing people's attention*.” (Health Expert, ID_02)  *“*[I hear] *I don't look like a stroke patient. Why did this happen to me?”* (Health Expert, ID_02) |
|  | - Impact of stroke | “*When people think of stroke, we just think of the medical impact only. But there's a lot more going on around it, socio-cultural aspects*” (Community, ID_17)  *“The stroke doesn't just impact the person who's had the brain injury, but it also impacts the actual family, loved ones, parents, children, friends”* (Community, ID_12)  “*I'm big on the fact that the impact of stroke must include stuff that's you know, unseen*” (Community, ID_12) |
|  | - Stroke is preventable | “*I guess discussion earlier, could it be prevented? That earlier intervention, whether it's from the GP* [general practitioner] *or from the local community*.” (Health Expert, ID_05)  “*Could also include some 'success stories' about how people have managed to reduce their risk*” (Health Expert, ID_09)  *“In terms of that prevention, it's really simple and that it's really achievable. We don't have to do drastic things to prevent stroke.”* (Community, ID_13) |
|  | - Secondary benefits | “*I also think that this program could be useful to the family members of people who had a stroke as far as educating themselves in what's happened today to their loved one... It's a broader education of what strokes are all about*.” (Community, ID_15) |
|  | **Empowerment & agency**   - Knowledge is power | *“It's important to become an informed patient. So how do you do that? You talk to people who had strokes, other people who had similar lived experiences, that sort of stuff, and basically get as much information as you can.”* (Community, ID_21) |
|  | - Ability to make choices and change | *“Is there potential to provide participants with a "GP* [general practitioner] *checklist" that they can use to jot down questions in advance? The idea of prioritising the list also gives the person 'agency'”* (Health Expert, ID_02)  *“*[it’s important] *people are given a sense that they can apply that information, and, you know, proceed as an almost an action plan.”* (Health Expert, ID_09) |
| ***Interactive environment*** *(related to the specific content, instruction, assessment & target audience)*  ***Interactive environment cont’d*** | **Presentation modalities**   - Simplicity in presentation | *“I agree that the music was too much for me as well. I found that really distracting. Some of the other videos had American accents which I didn't engage with very much.”* (Community, ID_18)  “*Keeping it in, you know simple language, jargon-free, dot points, key messages. All those kind of good health literacy practices* [are important].” (Health Expert, ID_09) |
|  | - Communication style | *“It's very easy to understand and just having someone to explain it rather than just reading a few dot points on the, whereas I think the video format is definitely good.”* (Community, ID_17)  *“So maybe a combination of an expert plus somebody was lived experience.”* (Health Expert, ID_09)  *“Different modalities of information delivery [are good], e.g. some short videos or vox-pops, animations.”* (Health Expert, ID_09) |
|  | - Functionality considerations | *“Like a little record, like a little badge or something when they're done, like a gold star or something like that.”* (Health Expert, ID_06) |
|  | **Content**   - Accuracy of information | “[Need to be careful].. *I think some of the information was also inaccurate (maybe they were bending the facts a little for ease of explanation).”* (Health Expert, ID_04)  “*It's misleading to a certain extent, it's simple but misleading, because my understanding of getting a cholesterol check involves fasting, then testing your blood, then it goes to pathology, then it comes back. So that's not a 20 min exercise*.” (Community, ID_21) |
|  | - Ease of understanding | *“Lay language is really important for health literacy.”* (Health Expert, ID_03)  *“It needs to be visually appealing and easy to understand, especially for the older generation”* (Health Expert, ID_09) |
|  | - Important topics of focus | “*High blood pressure message could have a little 'action' statement, like see your GP* [general practitioner] *to check your blood pressure*.” (Health Expert, ID_09) |
|  | **Personalisation/tailoring**   - Relatable information | “*Everyone is different and has different needs – it’s important to focus on making information as personal as possible.”* (Health Expert, ID_06) |
|  | - Experience to suit personal needs | *“There may be an additional module that goes into improving health literacy around prevalence, recurrence, policy, that sort of thing. You can choose to take that. But it's actually not going to be part of the core.”* (Health Expert, ID_08) |
|  | **Opportunities for wider learning**   - Generation of own knowledge | “*I need to do this, this and this as a sort of a next step, almost like a directory, even if it's as simple as you know, find a GP* [general practitioner]*… If there are services that we're encouraging people to access, is there a way that we could direct them straight from the platform.*” (Health Expert, ID_10) |
|  | - Social connectivity & self-reflection | *“Knowledge checks throughout work well.”* (Health Expert, ID_02)  *“I think a message that asks you to interact is actually quite helpful. Just from experience. Recently, through an insurance chat function. That automated you know, chat box type stuff where you have to interact. And it suggests some answers. It's just kind of giving you some feedback around engagement as well, so that could be helpful.”* (Health Expert, ID_02) |
| ***Technology infrastructure*** *(accessibly & inclusivity)* | **Accessibility**   - Compatibility with different technologies and devices | *“So* [it needs to be compatible] *across different technologies, ….. on an apple phone or an android, whether you're doing it on your ipad at night, or you're doing it on your computer during work hours….”* (Health Expert, ID_08)  “…*it’s also about the technical support, if they need help doing something, especially with the MOOC, there might be glitches*.” (Health Expert, ID_08) |
|  | **Inclusivity**   - Ability to cater to learning needs of diverse populations | *“As a deaf person (I hear via bi-lateral cochlear implants and assistive technology) I have no discretion on the sounds entering my brain, so no music in the background just one person speaking at a time.”* (Community, ID_21)  “*Colour and contrast – it may pay to liaise with Vision Australia who are experts dealing with people with vision impairment such as pigmenta retinosa, low vision, or colour blindness. There may be a need to alter contrast or different hatching rather than colour to discern areas*.” (Community, ID_21)  “*Hyperlink to Stroke Foundation's linguistic resources for F.A.S.T. Signs of stroke are really helpful, making it accessible for people from diverse cultural backgrounds.”* (Community, ID_17) |
| ***Evidence-based improvement*** *(ongoing assessment and improvements to the learning environment)* | **Ongoing improvement and testing** | *“…all the people with aphasia were good speakers, and not everyone who has the aphasia can speak as fluently as some of those people. So maybe it would have been nice to have somebody there who struggles a little bit to speak as fluently as those people did*.” (Community, ID_20)  “*I think that is common for people with the aphasia, but also I thought that could also be useful for linguistically diverse people as well, they might find it easier if most of the key points were bolded*.” (Community, ID_20) |

Adapted from the Framework for the Design & evaluation of MOOCs (Grover et al., 2013)

**COREQ (COnsolidated criteria for REporting Qualitative research) Checklist**

Developed from: Tong A, Sainsbury P, Craig J. Consolidated criteria for reporting qualitative research (COREQ): a 32-item checklist for interviews and focus groups. International Journal for Quality in Health Care. 2007;19(349 – 357)

| **No.** | **Item** | **Guide questions/ description** | **Page** |  |
| --- | --- | --- | --- | --- |
| **Domain 1: research team and reflexivity** | | | | |
| Personal Characteristics | | | | |
| 1 | Interviewer/facilitator | Which author/s conducted the interview or focus group? | 3 |  |
| 2 | Credentials | What were the researchers credentials? *E.g. PhD, MD* | 3 |  |
| 3 | Occupation | What was their occupation at the time of the study | 3 |  |
| 4 | Gender | Was the researcher male or female? | 3 |  |
| 5 | Experience and training | What experience or training did the researcher have | 3 |  |
| Relationship with participants | | | |  |
| 6 | Relationship established | Was a relationship established prior to study commencement? | 3 |  |
| 7 | Participant knowledge of the interviewer | What did the participants know about the researcher? *E.g. personal goals, reasons for doing the research* | 3 |  |
| 8 | Interviewer characteristics | What characteristics were reported about the interviewer/facilitator? *E.g Bias, assumptions, reasons and interests in the research topic* | 3 |  |
| **Domain 2: study design** | | | |  |
| Theoretical framework | | | |  |
| 9 | Methodological orientation and theory | What methodological orientation was stated to underpin the study? *E.g. grounded theory, discourse analysis, ethnography, phenomenology, content analysis* | - |  |
| Participant selection | | | |  |
| 10 | Sampling | How were participants selected? *E.g. purposive, convenience, consecutive, snowball* | 3 |  |
| 11 | Methods of approach | How were participants approached? *E.g. face-to-face, telephone, mail, email* | 3 |  |
| 12 | Sample size | How many participants were in the study? | 9 |  |
| 13 | Non-participation | How many people refused to participate or dropped out? Reason? | 3 |  |
| Setting | | | |  |
| 14 | Setting of data collection | Where was the data collected? *E.g. home, clinic, workplace* | 3 |  |
| 15 | Presence of non-participants | Was anyone else present besides the participants and researchers? | 3 |  |
| 16 | Description of sample | What are the important characteristics of the sample? *E.g. demographic data, date* | 9 & Supplemental Table 1 |  |
| Data collection | | | |  |
| 17 | Interview guide | Were questions, prompts, guides provided to the authors? Was it pilot tested? | 5-8 |  |
| 18 | Repeat interviews | Were repeat interviews carried out? If yes, how many? | 8 focus groups with each group |  |
| 19 | Audio/visual recording | Did the research use audio or visual recording to collect the data? | 3 |  |
| 20 | Field notes | Were field notes made during and/or after the interview of focus group | 3 |  |
| 21 | Duration | What was the duration of the interviews or focus group? | 3 |  |
| 22 | Data saturation | Was data saturation discussed? | 26 |  |
| 23 | Transcripts returned | Were transcripts returned to participants for comment and/or correction? | No, but live summaries included during focus groups |  |
| **Domain 3: analysis and findings** | | | |  |
| Data analysis | | | |  |
| 24 | Number of data coders | How may coders coded the data? | 8 |  |
| 25 | Description of the coding tree | Did authors provide a description of the coding tree? | 8-9, 11-14 |  |
| 26 | Derivation of themes | Were themes identified in advance or derived from the data? | 8-9 |  |
| 27 | Software | What software, if applicable, was used to manage the data? | 8 |  |
| 28 | Participant checking | Did participants provide feedback on the findings? | Yes, provided feedback on developed platform |  |
| Reporting | | | |  |
| 29 | Quotations presented | Were participant quotations presented to illustrate the themes/findings? Was each quotation identified? *E.g. participant number* | Throughout results |  |
| 30 | Data and findings consistent | Was there consistency between the data presented and the findings? | Throughout results |  |
| 31 | Clarity of major themes | Were major themes clearly presented in the findings? | Throughout results |  |
| 32 | Clarity of minor themes | Is there a description of diverse cases or discussion of minor themes? | Throughout results |  |
